# Transglutaminase 2 is associated with adverse colorectal cancer survival and represents a therapeutic target

**DOI:** 10.1038/s41417-023-00641-y

**Published:** 2023-07-13

**Authors:** Patrizia Malkomes, Ilaria Lunger, Elsie Oppermann, Johannes Lorenz, Sara Fatima Faqar-Uz-Zaman, Jiaoyan Han, Sabrina Bothur, Paul Ziegler, Katrin Bankov, Peter Wild, Wolf Otto Bechstein, Michael A. Rieger

**Affiliations:** 1 Department for General, Visceral, Transplant and Thoracic Surgery, Goethe University, Frankfurt am Main, Germany; 2 Frankfurt Cancer Institute, Frankfurt am Main, Germany; 3 German Cancer Consortium (DKTK) and German Cancer Research Center (DKFZ), Heidelberg, Germany; 4 Department of Medicine II, Hematology/Oncology, Goethe University, Frankfurt am Main, Germany; 5 Dr. Senckenberg Institute of Pathology, Goethe University, Frankfurt am Main, Germany; 6 University Cancer Center (UCT), Frankfurt am Main, Germany; 7 Cardio-Pulmonary-Institute, Frankfurt am Main, Germany

**Keywords:** Colorectal cancer, Biomarkers

## Abstract

Molecular markers for predicting prognosis of colorectal cancer (CRC) patients are urgently needed for effective disease management. We reported previously that the multifunctional enzyme Transglutaminase 2 (TGM2) is essential for CRC cell survival by inactivation of the tumor suppressor p53. Based on these data, we determined the clinical relevance of TGM2 expression and explored its potential as prognostic marker and therapeutic target in CRC. We profiled TGM2 protein expression in tumor samples of 279 clinically characterized CRC patients using immunohistochemical staining. TGM2 expression was upregulated in matched tumor samples in comparison to normal tissue. A strong TGM2 expression was associated with advanced tumor stages and predicted worse prognosis regarding progression-free and overall-survival, even at early stages. Inhibition of TGM2 in CRC cell lines by the inhibitors LDN27219 and Tyrphostin resulted in a strong reduction of cancer cell proliferation and tumorsphere formation in vitro by induction of p53-mediated apoptosis. Primary patient-derived tumorsphere formation was significantly reduced by inhibition of TGM2. Treatment of mice with TGM2 inhibitors exhibited a significant deceleration of tumor progression. Our data indicate that high TGM2 expression in CRC is associated with worse prognosis and may serve as a therapeutic target in CRC patients with strong TGM2 expression.

## Introduction

Globally, colorectal cancer (CRC) was responsible for 1.88 million new cases and 915,000 deaths in 2020, making it the third most common cancer and the second leading cause of cancer-related death [1]. To date, decision making regarding the treatment for CRC mainly relies on the tumor-node-metastasis (TNM) staging. Patients with localized stage should undergo surgical resection as a curative treatment. In patients with high-risk clinicopathological features adjuvant chemotherapy is advisable to prevent disease relapse [2, 3]. Despite remarkable improvements of therapeutic regimens and surgical techniques, recurrence rates are still high and resistance to chemotherapy often occurs. There is an urgent need to improve selection of early-stage patients who may benefit from adjuvant therapy and to identify patients with metastasis who may benefit from targeted therapies. Currently, available biomarkers for predicting the individual prognosis and therapeutic response of patients with CRC have limited accuracy. Mutations in *RAS (rat sarcoma virus)* and the mismatch repair gene deficiency, are two biomarkers that are routinely tested in clinics for risk stratification and for the choice of the best therapeutic regimen [4]. Identifying new biomarkers that can reliably classify CRC patients and that may serve as novel therapeutic targets remains a research priority.

Transglutaminase 2 (TGM2) is a member of the transglutaminase family of enzymes that catalyzes calcium-dependent posttranslational modification of proteins [5]. In addition to the transamidase activity, TGM2 can catalyze calcium-independent activities, such as GTP/ATP hydrolyse [6–8]. TGM2 is involved in the regulation of diverse physiological processes such as cell death, survival and adhesion [9, 10]. A biological role of TGM2 in cancer cell survival, metastasis and invasion was reported [11–13]. A variety of TGM2 inhibitors have been identified and their application to mouse models has shown therapeutic potential, e.g., in Huntington’s Disease [14]. There are no reports determining the effects of TGM2 inhibitors in preclinical models of CRC. Our previous study identified TGM2 as a fundamental survival factor in CRC cells by a direct inhibition of the tumor suppressor p53. We confirmed an elevated expression of TGM2 in tumor tissues compared with matched normal colon epithelium and highlighted TGM2 as a potential target for the diagnosis and treatment of CRC [15].

Several reports have demonstrated elevated expression of TGM2 in various cancer entities, and a correlation of strong TGM2 expression and poor prognosis has been shown [16, 17]. In CRC, an increased TGM2 mRNA expression in tumor tissue in comparison to normal tissue was reported with an association to a worse overall survival [18, 19]. However, the exact significance of TGM2 in CRC progression has not yet been elucidated.

In the present study, we clarify the clinical significance of TGM2 in CRC by evaluating the correlation of TGM2 protein expression and clinicopathological characteristics in a comprehensive CRC patient cohort. Further, we determined the effect of TGM2 inhibitors in preclinical CRC models.

## Materials and Methods

Detailed descriptions regarding the experimental procedures are provided in the Supplementary Material.

### Clinical tissue samples

A total of 279 patients with CRC were enrolled and underwent surgery at the University Hospital Frankfurt between 2008 and 2018. Tumor tissue samples were obtained from the biobank of the Goethe University Frankfurt Cancer Center’s Tissue Procurement Facility. The study was approved by the institutional ethics review board (Number: SGI-04-2014). Written informed consent was obtained from all participants prior to inclusion in the study.

### Immunohistochemistry (IHC) of human tissue microarrays (TMA)

The construction of the human TMAs was performed as described before [20].

To assess TGM2 expression in tumor and adjacent normal colon mucosa, immunohistochemical stainings on the constructed TMAs were performed, as described previously [15]. Each TMA was stained by using anti-TGM2 antibody (1:100, clone CUB7402, Abcam, Cambridge, UK) and anti-p53S15 antibody (1:100, #AF1043, R&D Systems, Minneapolis, USA). The specificity of the monoclonal TGM2 antibody has been confirmed before [15]. To confirm the specificity of the antibody against p53(S15) we stained wild type HCT-116 colon cancer cells as well as p53 knockdown HCT-116 cells. There was a specific nuclear p53(S15) staining in wild type cells, while p53 knockout cells showed no staining. (Supplementary Fig. S1). Further, an isotype control was used to exclude unspecific antibody binding.

TGM2 staining scores were determined independently by two investigators. Investigators were blinded to tumor stage and patient outcome.

For correlation analysis, staining scores were summarized as weak (total score 0–6), moderate (total score 7–11) and strong expression (total score 12–15).

### Isolation of primary cells from patient specimens

Fresh colon cancer tissue was obtained from patients undergoing surgical resection between 2014–2016 at Goethe University Hospital Frankfurt or at Bethanien-Hospital (Frankfurt, Germany), who had given informed consent. Isolation of primary CRC cells was performed according to a previously published method [21].

### In vivo xenograft experiments

All animal experiments were performed according to protocols approved by the legal state authorities. Female NOD.CB17-*Prkdc*^*scid*^/J (NOD-SCID) mice (Jackson Laboratory, Maine, USA) at 6–8 weeks of age were used for experiments. LDN27219 and Tyrphostin 47 (both Sigma Aldrich, St. Louis, USA) were dissolved in dimethyl sulfoxide (DMSO).

A total of 5 × 10^4^ living SW480 cells were subcutaneously injected into the flank of NOD-SCID mice. When tumor size reached 0.2–0.3 cm in diameter, LDN27219 (25 mg/kg) was administered orally and Tyrphostin 47 (Tyrphostin) (2.2 mg/kg) intraperitoneally and compared with a DMSO control. Six mice were allocated per treatment and control group, respectively. Tumor growth was measured twice weekly using a caliper. The investigator was not blinded to group allocation. Tumors were harvested and lysates were prepared for transglutaminase activity assay and protein expression analysis by Simple western technology.

## Results

### TGM2 protein expression in CRC tissue samples

**Fig. 1:**
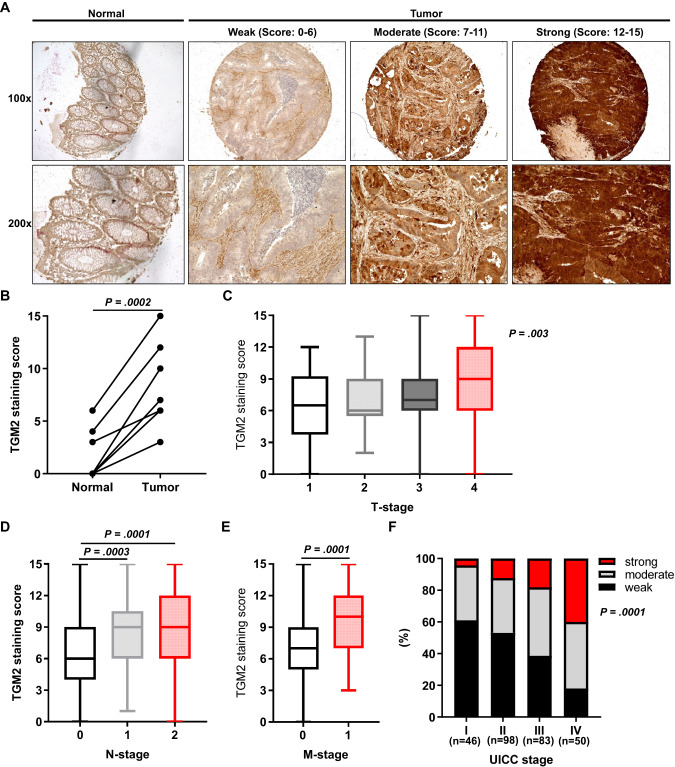
TGM2 is elevated in colon cancer tissue and is correlated with advanced tumor stages. **A** Representative photomicrographs (magnification = 100× and 200×) of immunohistochemical staining for TGM2 (brown) expression in tumor and normal tissue from CRC patients representing weak, moderate and strong expressing tumors. Expression levels were determined in epithelial cells by two independent investigators using a three-stage staining score. **B** TGM2 staining score in matched normal and tumor tissue of 7 CRC patients. **C** Mean TGM2 staining score according to T-stage, **D** N-stage, and **E** M-stage. Data are presented as mean immunoreactive score ± SD of IHC staining. Significance was determined using Wilcoxon matched pairs or Kruskal-Wallis test. **F** Prevalence of weak, moderate or strong TGM2 expression in 279 CRC patients according to their UICC stages. Correlation was calculated using Spearman correlation.

We assessed TGM2 protein expression via immunohistochemical stainings on samples of 279 clinically well-characterized CRC patients, which have entered the University Hospital Frankfurt between 2008 and 2018. We determined the intensity of TGM2 protein expression in epithelial cells of the tumor. Of the 279 CRC specimens, 44.1% were categorized into the weak, 37.6% into the moderate and 18.3% into the high expression group (Table 1). Representative microphotographs of different TGM2 expression levels are shown in Fig. 1A. TGM2 was expressed to a higher extent in epithelial cancer tissue than in matched normal colon epithelium in all investigated cases (Fig. 1B). Strong TGM2 expression was associated with tumor size (Fig. 1C), lymph node metastasis (Fig. 1D) and distant metastasis (Fig. 1E). Moreover, a strong TGM2 expression was more frequent in patients with advanced tumor stages (Fig. 1F).

**Table 1:** Patient and tumor characteristics in correlation to TGM2 expression.

Characteristic	Total cohort	TGM2 expression			*P*-value
	(*n* = 279)	Weak (*n* = 123)	Moderate (*n* = 105)	Strong (*n* = 51)	
Mean age ± SD (years)	67.9 (12.3)	66.4 (12.9)	70.5 (11.6)	65.9 (11.2)	**0.023**
Sex					0.911
Male	156 (55.9)	67 (54.5)	60 (57.1)	29 (56.9)	
Female	123 (44.1)	56 (45.5)	45 (42.9)	22 (43.1)	
Charlson comorbidity index					0.101
<3	100 (35.8)	46 (37.4)	39 (37.1)	15 (29.4)	
3–4	69 (24.7)	38 (30.9)	20 (19.0)	11 (21.6)	
>4	110 (39.4)	39 (31.7)	46 (43.8)	25 (49.0)	
Tumor location					0.229
Ascending colon	134 (48.0)	66 (53.7)	48 (45.7)	20 (39.2)	
Descending colon	37 (13.3)	16 (13.0)	11 (10.5)	10 (19.6)	
Sigmoid	108 (38.7)	41 (33.3)	46 (43.8)	21 (7.5)	
Tumor differentiation					0.183
Well to moderate	238 (86.9)	105 (86.8)	94 (90.4)	39 (79.6)	
Poor	36 (13.1)	16 (13.2)	10 (9.6)	10 (20.4)	
Tumor depth					0.157
T1	13 (4.7)	7 (5.7)	5 (4.8)	1 (2.0)	
T2	45 (16.1)	25 (20.3)	15 (14.3)	5 (9.8)	
T3	169 (60.6)	75 (61.0)	64 (61.0)	30 (58.8)	
T4	52 (18.6)	16 (13.0)	21 (20.0)	15 (29.4)	
Lymph node status					**<0.001**
N0	154 (55.6)	84 (68.9)	52 (50.0)	18 (35.3)	
N1	60 (21.7)	17 (13.9)	31 (29.8)	12 (23.5)	
N2	63 (22.7)	21 (17.2)	21 (20.2)	21 (41.2)	
Distant metastasis					**< 0.001**
M0	227 (82.2)	111 (91.7)	85 (81.0)	31 (62.0)	
M1	49 (17.8)	10 (8.3)	20 (19.0)	19 (38.0)	
Lymphatic invasion					**0.017**
L0	154 (57.7)	74 (64.9)	60 (57.7)	20 (40.8)	
L1	113 (42.3)	40 (35.1)	44 (42.3)	29 (59.2)	
Venous invasion					**0.030**
V0	238 (88.8)	103 (90.4)	95 (92.2)	40 (78.4)	
V1	30 (11.2)	11 (9.6)	8 (7.8)	11 (21.6)	
Perineural invasion					0.078
Pn0	211 (87.9)	96 (92.3)	81 (87.1)	34 (79.1)	
Pn1	29 (12.1)	8 (7.7)	12 (12.9)	9 (20.9)	
UICC stage					**<0.001**
I	45 (16.1)	29 (23.6)	13 (12.4)	3 (5.9)	
II	101 (36.2)	54 (43.9)	34 (32.4)	13 (25.5)	
III	83 (29.7)	30 (24.4)	38 (36.2)	15 (29.4)	
IV	50 (17.9)	10 (8.1)	20 (19.0)	20 (39.2)	
Tumor relapse/progress					< **0.001**
No	175 (69.2)	100 (39.3)	61 (64.9)	14 (29.8)	
Yes	78 (30.8)	12 (10.7)	33 (35.1)	33 (70.2)	
Mean CEA					0.094
Mean ± SD (ng/ml)	438 (4998)	9 (22)	218 (1752)	2028 (11779)	
Adjuvant therapy	87 (32.7)	27 (23.1)	36 (35.3)	24 (51.1)	**0.002**
p53 status					**0.009**
Wildtype	133 (51.2)	70 (61.9)	44 (43.6)	19 (41.3)	
Mutant	127 (48.8)	43 (38.1)	57 (56.4)	27 (58.7)	
Kras status					**0.041**
Wildtype	29 (31.2)	7 (17.5)	12 (38.7)	10 (45.5)	
Mutant	64 (68.8)	33 (82.5)	19 (61.3)	12 (54.5)	

*NOTE*: Values are No. (%) unless otherwise indicated. For analysis of categorical variables Chi-square test was used. Fishers exact test was used, if appropriate. For continuous variables one-way ANOVA analysis with Bonferroni correction was performed. *UICC* International Union Against Cancer, *CEA* Carcinoembryonic antigen, *TGM2* Transglutaminase 2.

Bold values indicates statistical significant P values (P < 0.05).

### Correlations of TGM2 level with clinicopathological characteristics in CRC

Patient and histopathological characteristics according to TGM2 expression are listed in Table 1. Patients undergoing adjuvant chemotherapy and patients developing tumor relapse showed more frequently a strong TGM2 expression in their primary tumor. However, no associations were discovered between TGM2 expression level and other clinical factors, including gender, comorbidities, or tumor location.

Our correlation analysis of TGM2 with histopathological factors revealed that a strong TGM2 expression was strongly associated with higher N stage (*p* < 0.001), M stage (*p* < 0.001) and International Union Against Cancer stage (UICC, *p* < 0.001) as well as with a lymphatic and venous invasion. Interestingly, mutations in TP53 correlated with a strong TGM2 expression, while tumors with a Kras mutation showed predominantly weak TGM2 expression.

### Prognostic significance of TGM2 expression for overall and disease-free survival

**Fig. 2:**
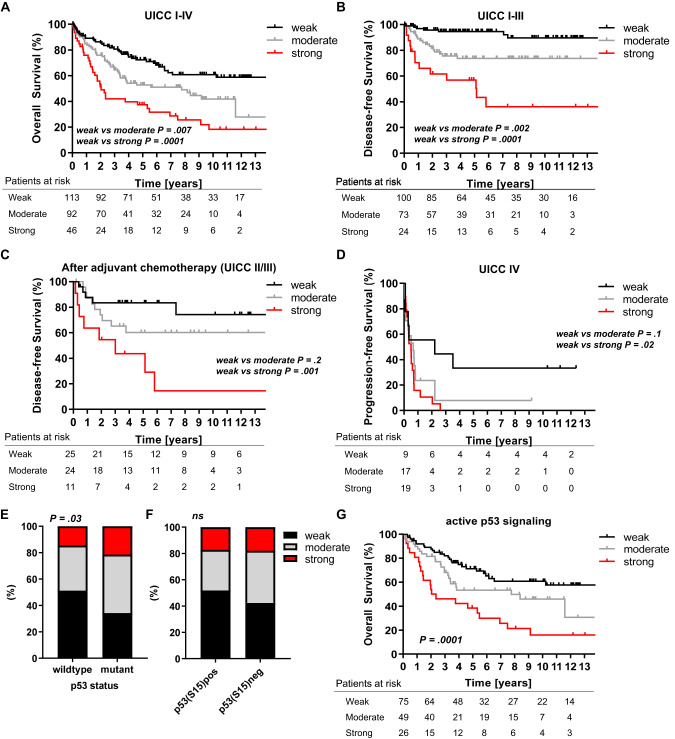
Strong TGM2 expression predicts poor outcome in CRC patients. **A** Kaplan-Meier analysis of overall survival in all CRC patients with weak, moderate or strong TGM2 expression, assessed by IHC. **B** Kaplan-Meier analysis of disease-free survival in CRC patients after curative tumor resection with weak, moderate or strong TGM2 expression. Patients with UICC stage IV were excluded. **C** Kaplan-Meier analysis of disease-free survival for patients with UICC stage II and III after adjuvant chemotherapy according to weak, moderate or strong TGM2 expression. **D** Kaplan-Meier analysis of progression-free survival assessed by imaging in patients with UICC stage IV according to TGM2 expression. Significance was calculated by log-rank test. **E** Prevalence of weak, moderate or strong TGM2 expression in p53 wildtype and p53 mutant tumors. **F** Prevalence of weak, moderate or strong TGM2 expression in p53(S15) positive and p53(S15) negative tumors. **G** Kaplan-Meier analysis of overall survival in CRC patients with active p53 signaling (p53 wildtype and p53 mutant tumors with positive p53(S15) staining) according to TGM2 expression. “Patient at risk” indicate the number of patients who had not yet experienced the event of interest (death, disease-relapse or disease-progression) in each group (TGM2 expression weak, moderate or strong) at the corresponding time point (starting point, 2, 4, 6, 8, 10, and 12 years of follow-up). Significance was calculated by log-rank test.

We compared postoperative survival and recurrence according to TGM2 expression levels in our patient cohort. As expected, UICC stage IV correlated with a shorter overall survival (OS), whereas patients at UICC stage I–III demonstrated similar long-term OS (Supplementary Fig. 2). Kaplan–Meier-survival analyses showed a significantly reduced OS of patients with strong TGM2 expression compared to the moderate and weak expression group, also in subgroup analysis of patients with UICC I–III (Fig. 2A, Supplementary Fig. 3). Importantly, when we determined the disease-free survival (DFS) of patients after curative tumor resection, we could separate the patients using the TGM2 expression level at diagnosis: patients with strong TGM2 expression developed tumor recurrence more frequently than patients with weak or moderate TGM2 staining index (Fig. 2B, *p* = 0.0001). Similar, DFS was significantly worse in TGM2 strong-expressing patients at UICC stage II or III, regardless of whether patients received adjuvant chemotherapy after surgical resection or not (Fig. 2C, Supplementary Fig. 4). Patients diagnosed with stage IV CRC presenting with high TGM2 expression levels had a lower progression-free survival (PFS) than patients with lower TGM2 expression (Fig. 2D). Here, PFS was defined as time from treatment to disease progression in patients who still showed measurable tumor lesions after tumor resection. Importantly, the level of TGM2 expression was inversely correlating with the OS and recurrence-free survival, suggesting a dose-dependency of TGM2 (Fig. 2A–D).

The univariate Cox regression analysis indicated that age (HR 1.05, *p* < 0.001), UICC stage IV (HR 4.05, *p* < 0.001) and strong TGM2 expression (HR 2.44, *p* < 0.001) were negative predictors for OS. Results from multivariate analysis after adjustment revealed that strong TGM2 expression is an independent prognostic factor for OS (HR 3.036, *p* = 0.007), alongside with patient age (HR 1.05, *p* = 0.004) at a much lower HR (Supplementary Table 1).

Cox regression analysis for factors influencing DFS is shown in Supplementary Table 2. Univariate analysis revealed following factors as significant negative predictors for DFS: lymph node metastasis (HR 3.291, *p* = 0.001), UICC stage III (HR 9.446, *p* = 0.002), Kras mutation (HR 8.938, *p* < 0.001) and strong TGM2 expression (HR 10.396, *p* < 0.001). Multivariate analysis further demonstrated strong TGM2 expression (HR 15.87, *p* = 0.021) and lymph node metastasis (HR 13.16, *p* = 0.021) as independent predictors for tumor relapse.

### Association of TGM2 expression and p53 status and activity

We found that tumors with a p53 mutation show more frequently a strong TGM2 expression than p53 wildtype tumors (Fig. 2E). Further, phosphorylated p53 (p53S15) was predominantly observed in tumors with weak TGM2 expression (51.9%) and not in TGM2 strong expressing tumors (17.3%) (Fig. 2F). Last, in our patient cohort the p53 mutational status or the presence of phosphorylated p53 had no influence on OS (Supplementary Fig. 5A, B). Considering the p53 activity status, strong TGM2 expression in patients with measurable p53 signaling (p53 wildtype and mutated tumors with positive p53(S15) staining) again predicted worse OS (Fig. 2G). On the other hand, in patients without p53 activity (as measured by phosphorylated p53(S15)) TGM2 expression levels were not associated with OS, suggesting a dependency of TGM2 on p53 signaling (Supplementary Fig. 6).

### Inhibition of TGM2 results in a reduction of CRC cell proliferation and tumorsphere formation by induction of p53-mediated apoptosis

**Fig. 3:**
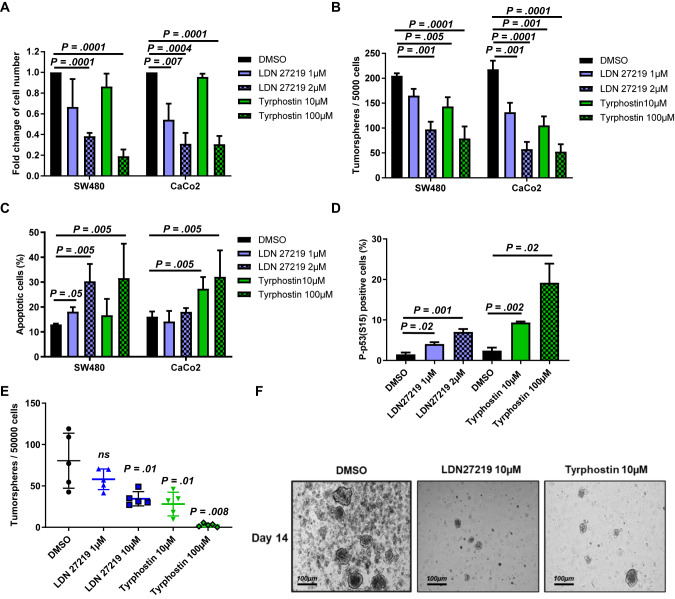
Inhibition of TGM2 reduces patient-derived tumorsphere formation in vitro by an induction of p53-mediated apoptosis. **A**–**D** SW480 and CaCo2 cells were treated with LDN27219 (1 µM and 2 µM), Tyrphostin (10 µM and 100 µM) or DMSO and subsequently analyzed. **A** Cell expansion after treatment. Shown are the mean fold changes of cell number normalized to DMSO control 72 h after treatment. **B** Mean number of tumorspheres counted 7 days after treatment. **C** Percentage of apoptotic cells determined by Annexin V/7-AAD staining 72 h after treatment. **D** Mean number of p53(S15) positive SW480 cells detected by Flow cytometry. **E** Isolated tumor cells derived from freshly resected CRC patient specimens were treated with LDN27219, Tyrphostin or DMSO and the number of formed tumorspheres was determined at day 14 (*n* = 5 CRC patients). **F** Representative photomicrographs of tumorspheres. Scale bar, 100 µm. Data are expressed as mean ± SD of at least three independent experiments. Significance determined using Multiple t-test or Mann-Whitney-U test.

The effect of TGM2 inhibition by LDN27219 and Tyrphostin was assessed in CRC cell lines. Treatment with these inhibitors significantly decreased transamidase activity (Supplementary Fig. 7A). LDN27219 and Tyrphostin significantly reduced cell proliferation of SW480 and CaCo2 cells after 72 h of treatment (Fig. 3A). Furthermore, TGM2 inhibition strongly reduced tumorsphere formation in both cell lines (Fig. 3B).

To assess the cell fate upon TGM2 inhibition, we performed AnnexinV/7AAD staining. In SW480 cells, both inhibitors induced apoptosis in comparison to DMSO treated cells. In CaCo2 cells, a significant induction of apoptosis could only be detected after treatment with Tyrphostin (Fig. 3C). In order to confirm an activation of p53 signaling upon TGM2 inhibition, we performed immunohistochemical stainings and flow cytometric analysis for phosphorylated p53(S15) in SW480 cells treated with LDN27219 or Tyrphostin. We detected a significant increase of phosphorylated p53(S15) in SW480 cells treated with TGM2 inhibitors, both in immunohistochemical stainings (Supplementary Fig. 7B) and flow cytometry (Fig. 3D), suggesting an induction of p53-mediated apoptosis. To translate our results to primary CRC patient material, tumorsphere cultures derived from freshly isolated CRC patient tissue were treated with TGM2 inhibitors. The number and diameter of formed tumorspheres were significantly reduced after 14 days of treatment with LDN27219 and Tyrphostin compared with the DMSO control (Fig. 3E, F).

### Inhibition of TGM2 reduces tumor growth in vivo by an induction of p53 signaling

**Fig. 4:**
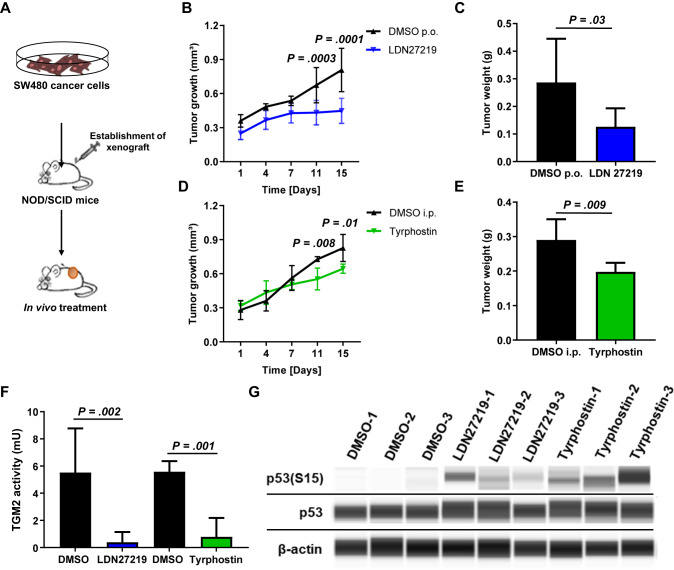
Inhibition of TGM2 reduces tumor growth in vivo by an induction of p53 signaling. **A** Schematic research setup. In total 5 × 10^4^ SW480 cells were injected subcutaneously into NOD-SCID mice. Once the tumor reached 20 mm^3^, mice were randomized into different groups (*n* = 6 mice per group) and treated with LDN27219 (25 mg/kg, three times/week), Tyrphostin (2.2 mg/kg, three times/week) or DMSO (control). **B** Tumor volumes under treatment with LDN27219 or DMSO over time. **C** Tumor weight after 3 weeks of treatment with LDN27219. **D** Tumor volumes under treatment with Tyrphostin or DMSO over time. **E** Tumor weight after 3 weeks of treatment with Tyrphostin. **F** Transamidation activity was assessed in explanted tumor xenografts using TGM2 activity assay. **G** Determination of p53 and phosphorylated p53(S15) protein expression in explanted tumor xenografts via Simple Western technology. β-actin served as loading control. See also Supplementary Fig. 8. Data are expressed as mean ± SD. Significance is determined using Multiple t-test or the Mann-Whitney-U test.

To demonstrate potential anticancer effects of TGM2 inhibition in a preclinical model we performed an in vivo treatment experiment. NOD/SCID mice with established SW480 tumor cell xenografts were treated with LDN27219, Tyrphostin, or DMSO control (Fig. 4A). While DMSO-treated mice showed a successive increase in tumor growth, LDN27219-treated mice showed a stagnation of tumor size, leading to small tumors that were as equal in size as before the start of treatment (Fig. 4B, C). Similarly, Tyrphostin treatment resulted in a significant inhibition of tumor progression (Fig. 4D, E). All treatment schedules were well tolerated and no reduction of mouse body weight or visible adverse effects have been observed. Analysis of explanted tumor xenografts demonstrated that a treatment with LDN27219 and Tyrphostin resulted in almost complete inhibition of transamidase activity in comparison to xenograft tumors derived from DMSO-treated mice (Fig. 4F). Similar to our in vitro data, we could detect an increase of phosphorylated p53(S15) in xenografts treated with TGM2 inhibitors in comparison to DMSO-treated xenografts, indicating an activation of p53 signaling through an inhibition of transamidase activity (Fig. 4G, Supplementary Fig. 8).

These results suggest that TGM2 inhibition may serve as a treatment strategy in CRC patients with high TGM2 expression.

## Discussion

Here we demonstrate TGM2 protein expression as a strong prognostic marker and as a potential target molecule for future therapeutic strategies in CRC. TGM2 not only functions as an indicator of poor prognosis for CRC patients, but also promotes the survival and growth of CRC cells. Analysis of 279 patients with CRC revealed that TGM2 is expressed in tumor cells in the majority of cases, and that almost 20% of patients show a strong TGM2 expression. These patients have a higher risk of recurrent disease and disease-associated death. An immunohistological staining can separate these patients according to the epithelial TGM2 expression. A previous study has shown that overexpression of TGM2 mRNA is associated with reduced OS in colon cancer [18]. Whether TGM2 was expressed in tumor epithelium or in the tumor microenvironment remained unclear. Further, stromal TGM2 expression was significantly associated with risk of recurrence in stage II patients [19]. This is the first study, that determined a significant correlation of epithelial TGM2 expression and advanced tumor stages in CRC. More important, we could demonstrate a statistical association of high TGM2 expression with poor survival and increased recurrence rates. Multivariate analyses identified strong TGM2 expression level as an independent prognostic factor for tumor-related mortality and tumor relapse. Moreover, the prognosis of advanced-stage CRC with strong TGM2 expression was significantly worse when patients were uniformly treated with oncologic tumor resection and adjuvant chemotherapy.

In our previous study, we identified TGM2 as a survival factor in CRC cells through inactivation of the tumor suppressor p53, thus the prosurvival function of TGM2 is dependent on the activity of p53 [15].

We demonstrated that a strong TGM2 expression in tumor was correlated with a more frequent mutation in the p53 gene. Interestingly, phosphorylated and thus activated p53 could mainly be detected in tumors with weak TGM2 expression, suggesting an inhibition of p53 activity by TGM2. Therefore, the p53 status of a tumor should be considered when evaluating the clinical significance of TGM2 expression. TGM2 strong expressing patients had shorter OS than TGM2 weak expressing patients, regardless of histological type. One of the possible explanations for this result is that a significant proportions of patients with p53 mutations show still p53 activity as demonstrated by our immunohistochemical stainings of phosphorylated p53S15. A total of 29.6% of p53 mutated tumors are positive for active p53. This is in concordance with the literature, showing that a relevant proportion of p53 mutations retain significant activity [22, 23]. Taking this into account, we could show that TGM2 only predicted poor OS in patients with active p53 signaling, but not in patients with mutant and inactive p53 signaling.

Another interesting aspect of TGM2 is its potential as a target for anticancer therapy. TGM2 enzymatic activity has been extensively studied, the crystal structure has been resolved [24], and specific small molecule inhibitors have been successfully translated into clinical trials for the treatment of celiac disease [25]. LDN2719 is a reversible, slow-binding TGM2 inhibitor, binding at the enzyme’s GTP site and locking it in its closed conformation thus inhibiting transamidase activity [26]. Tyrphostin is a kinase-related inhibitor that has been identified as a potent inhibitor of TGM2 activity [27]. We found that proliferation and tumorsphere formation in CRC cell lines and primary CRC cells were reduced by TGM2 inhibition using LDN27219 or Tyrphostin. Here, TGM2 inhibition results in an induction of p53-mediated apoptosis. Our preclinical mouse model shows that the application of TGM2 inhibitors leads to tumor growth inhibition of established xenograft tumors in vivo by an induction of p53 signaling. Our data are consistent with the tumor-promoting role of TGM2 observed in breast, ovarian and colon cancer cells. Previous studies have also identified inhibition of TGM2 as a potential target to enhance cancer cell death and chemosensitivity, e.g., in glioblastoma [28].

This study has several strengths. We tested the effects of TGM2 inhibitors in primary cultured CRC cells as well as in preclinical mouse models. We have shown that an inhibition of TGM2 transamidase activity by inhibitors is sufficient to reduce CRC cell proliferation and tumorsphere formation by an induction of p53-mediated apoptosis. This provides a rationale reason to utilize TGM2 inhibitors that block transamidase activity. Second, our immunohistochemical data revealed TGM2 as an independent predictor of poor prognosis and risk for recurrence, highlighting the clinical relevance of the present study.

This study has also some limitations. First, there are only two CRC cell lines that have been included. The functional role of TGM2 inhibitors has not been fully examined. Second, the clinical data of our patient cohort were collected retrospectively. However, our findings clearly indicated that patients with strong TGM2 expression are at risk for tumor relapse. We should develop new treatment strategies using known targeting agents or new targeting drugs for TGM2 strong expressing patients.

## Conclusion

Our results revealed that TGM2 may serve as a novel marker of prognosis in CRC. The inhibition of TGM2 suppressed CRC proliferation and tumor growth in vivo, suggesting that TGM2 may be a novel target in patients with strong TGM2 expression for clinical cancer therapy.

### Supplementary information

Supplementary Tables, Figures and Methods

## Data Availability

All data generated or analyzed during this study are included in the published article and its Supplementary information files.
