# Supplementary material for: Transglutaminase 2 is associated with adverse colorectal cancer survival and represents a therapeutic target
Source: Cancer Gene Ther. 2023 Jul 13;30(10):1346–54. doi: 10.1038/s41417-023-00641-y (PMC10581896; doi:10.1038/s41417-023-00641-y)
Supplement: Supplementary file 1 — Supplementary Tables, Figures and Methods [file 41417_2023_641_MOESM1_ESM.pdf]

Supplementary Table 1

Supp. Table 1. Cox proportional hazard model for factors influencing overall mortality								
**Factor**	**Univariate**				**Multivariate**			
	HR	95% CI	b-coefficient	P-value	HR	95% CI	b-coefficient	P-value
**Age (years)**	**1.054**	**1.035 – 1.073**	**0.052**	**<0.001**	**1.04**	**1.011 – 1.070**	**0.039**	**0.007**
Sex				0.948				
Male	1.012	0.705 – 1.452	0.012					
Lymph node metastasis				0.723				
N+	1.090	0.675 – 1.760	0.086					
UICC				**<0.001**				
IV	**4,046**	**2.251 – 7.271**	**1.398**					
**TGM2 expression**				**<0.001**				**0.007**
**Strong**	**2.444**	**1.542 – 3.874**	**0.894**		**3.036**	**1.359 – 6.785**	**1.111**	
CEA (ng/ml)	1.00	1.00 – 1.00	0	0.068				
adjuvant therapy				0.180				
Yes	0.760	0.508 – 1.136	-0.275					
p53 status				0.843				
mutated	1.039	0.713 – 1.512	0.038					
Kras status				**0.002**				0.620
mutated	**0.400**	**0.221 – 0.724**	**-0.916**		0.620	0.319 – 1.202	-0.479	

The univariate Cox regression model included age, sex, tumor stage, mutational status, CEA, TGM2 expression and adjuvant therapy. UICC-stage status was excluded in the multivariate Cox regression analysis to avoid multicollinearity. (See statistical analysis section for details).
Abbreviations: CEA, Carcinoembryonic antigen; CI, Confidence interval; HR, hazard ratio; TGM2, Transglutaminase 2; UICC, International Union Against Cancer

Supplementary Table 2

Supp. Table 2. Cox proportional hazard model for factors influencing tumor recurrence (without UICC IV)								
**Factor**	**Univariate**				**Multivariate**			
	HR	95% CI	b-coefficient	P-value	HR	95% CI	b-coefficient	P-value
Age (years)	1.008	0.981 – 1.036	0.008	0.562				
Sex				0.139				
Male	0.632	0.344 – 1.161	-0.459					
**Lymph node metastasis**				**0.001**				**0.021**
**N+**	**3.291**	**1.609 – 6.735**	**1.191**		**13.283**	**1.47 – 119.72**	**2.587**	
UICC				**0.002**				
III	**9.446**	**2.246 – 39.738**	**2.246**					
**TGM2 expression**				**<0.001**				**0.021**
**Strong**	**10.396**	**4.233 – 25.535**	**2.341**		**15.87**	**1.52 – 165.75**	**2.764**	
CEA (ng/ml)	1.020	0.993 – 1.047	0.019	0.145				
adjuvant therapy				**<0.001**				0.854
Yes	**3.716**	**1.996 – 6.919**	**1.313**		1.177	0.209 – 6.637	0.163	
p53 status				0.920				
mutated	1.032	0.555 – 1.921	0.032					
Kras status				**<0.001**				0.213
mutated	**8.938**	**3.107 – 25.709**	**2.190**		0.345	0.209 – 6.637	-1.064	

The univariate Cox regression model included age, sex, tumor stage, mutational status, CEA, TGM2 expression and adjuvant therapy. UICC stage status was excluded in the multivariate Cox regression analysis to avoid multicollinearity. (See statistical analysis section for details).
Abbreviations: CEA, Carcinoembryonic antigen; CI, Confidence interval; HR, hazard ratio; TGM2, Transglutaminase 2; UICC, International Union Against Cancer

Supplementary Figures

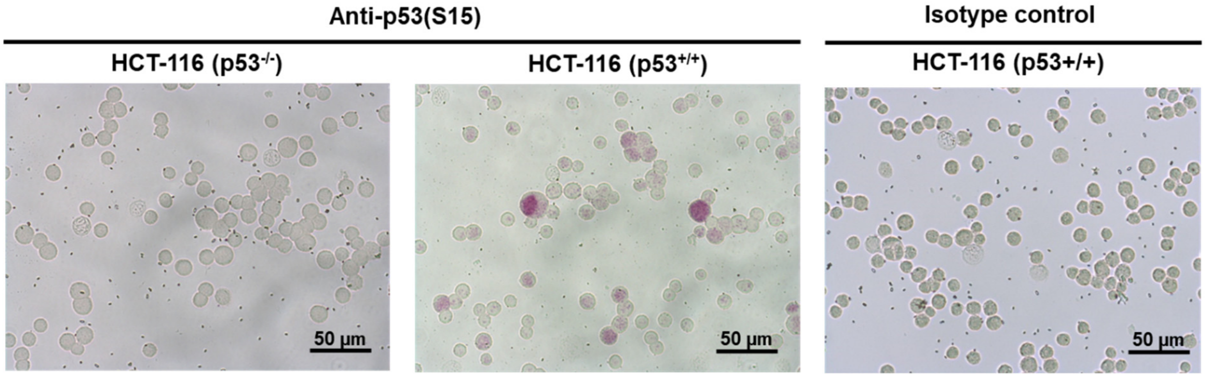

Supplementary Figure 1. Confirmation of antibody specificity against p53(S15). Representative microphotographs of HCT-116 cells after immunohistochemical (IHC) stainings of p53(S15) (red) or isotype control. Wild type HCT-116 ($p53^{+/+}$) or p53 knockout HCT-116 ($p53^{-/-}$) cells were stained to show specificity of anti-p53(S15) antibody used for IHC. Scale bar, 50 µm.

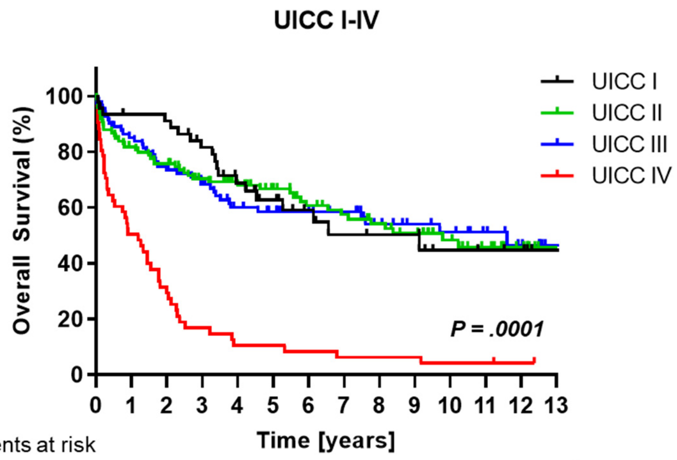

Patients at risk

UICC I	46	40	26	15	11	7	5
UICC II	99	75	56	41	33	21	9
UICC III	84	58	43	35	24	18	10
UICC IV	52	15	6	5	4	3	2

Supplementary Figure 2. Overall survival in the total CRC patient cohort. Kaplan-Meier analysis of overall survival in all CRC patients according to UICC tumor stages. Significance was calculated by log-rank test. „Patients at risk“ indicates the number of patients who are alive in each group (TGM2 expression weak, moderate or strong) at the corresponding time point (starting point, 2, 4, 6, 8, 10 and 12 years of follow up).

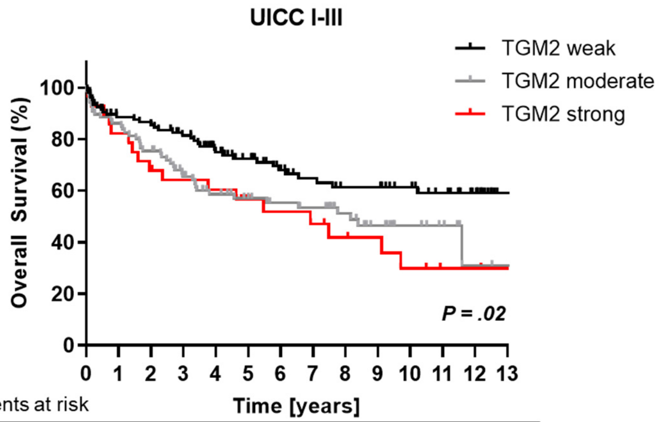

Patients at risk							
Weak	107	86	66	46	35	30	17
Moderate	87	65	41	32	23	10	3
Strong	28	20	17	12	9	6	4

Supplementary Figure 3. Strong TGM2 expression predicts poor overall survival in CRC patients. Kaplan-Meier analysis of overall survival in CRC patients at UICC stage I-III after curative tumor resection with weak, moderate or strong TGM2 expression, assessed by IHC. Significance was calculated by log-rank test. „Patients at risk“ indicates the number of patients who are alive in each group (TGM2 expression weak, moderate or strong) at the corresponding time point (starting point, 2, 4, 6, 8, 10 and 12 years of follow up).

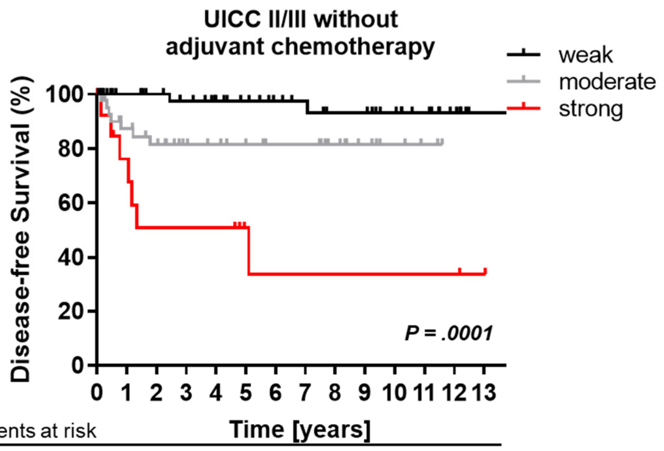

Patients at risk							
Weak	54	42	33	26	21	17	8
Moderat	45	29	21	16	12	5	1
Strong	14	7	7	3	3	3	3

Supplementary Figure 4. Strong TGM2 expression predicts poor disease-free survival in CRC patients. Kaplan-Meier analysis of disease-free survival in CRC patients at UICC stage II and III who did not receive adjuvant chemotherapy after curative tumor resection with weak, moderate or strong TGM2 expression. Significance was calculated by log-rank test. „Patients at risk“ indicates the number of patients who have not experienced tumor relapse in each group (TGM2 expression weak, moderate or strong) at the corresponding time point (starting point, 2, 4, 6, 8, 10 and 12 years of follow up).

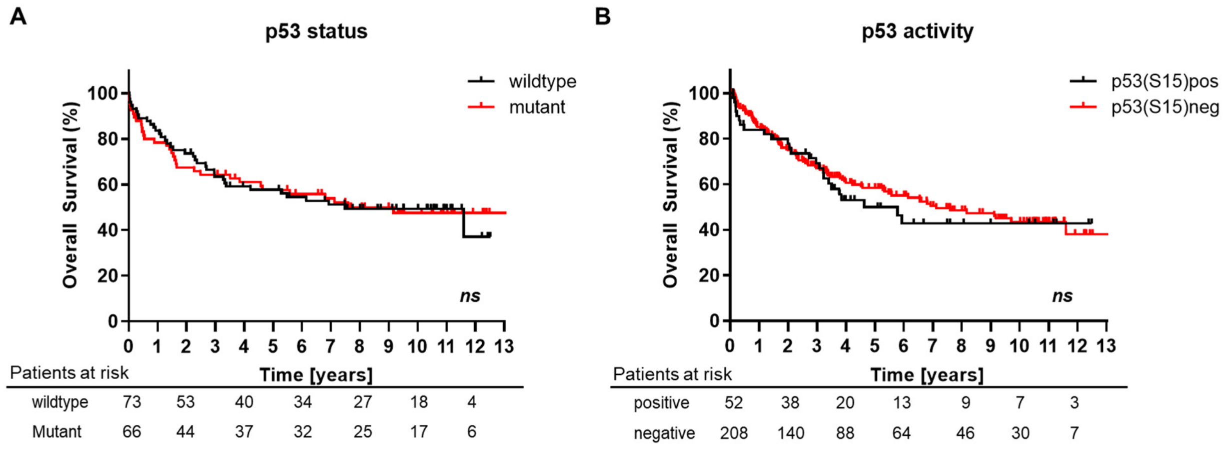

Supplementary Figure 5. Overall Survival according to p53 status and p53 activity. (A) Kaplan-Meier analysis of overall survival in CRC patients according to p53 mutational status. (B) Kaplan-Meier analysis of overall survival in CRC patients according to p53 activity assay, assessed by IHC staining for p53(S15). Significance was calculated by log-rank test. „Patients at risk“ indicates the number of patients who are alive in each group at the corresponding time point (starting point, 2, 4, 6, 8, 10 and 12 years of follow up).

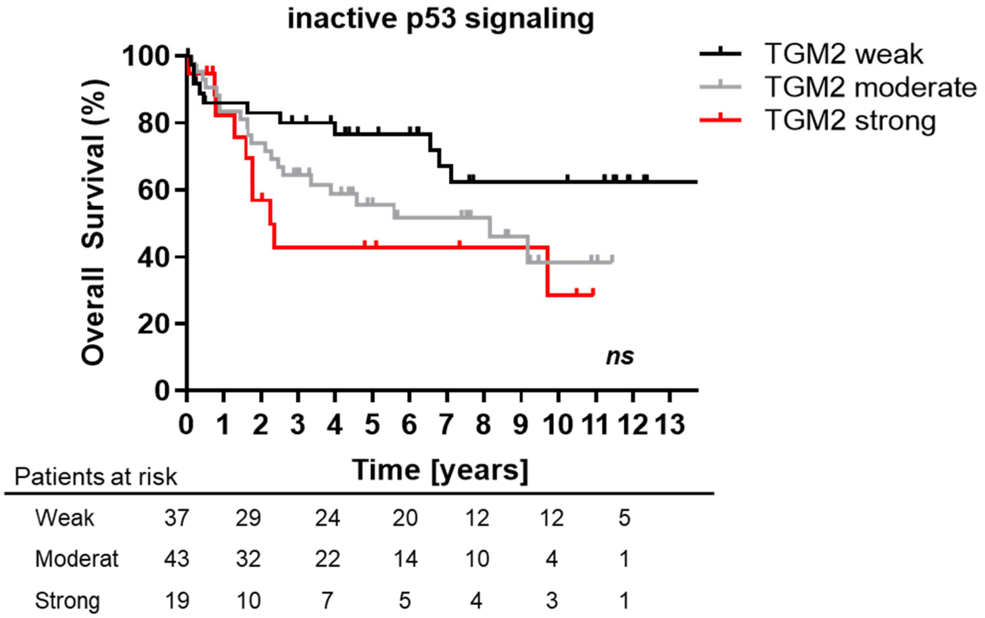

Patients at risk							
Weak	37	29	24	20	12	12	5
Moderat	43	32	22	14	10	4	1
Strong	19	10	7	5	4	3	1

Supplementary Figure 6. Overall Survival in CRC patients without p53 activity. Kaplan-Meier analysis of overall survival in CRC patients with inactive p53 signaling (as measured by phosphorylated p53(S15)) according to TGM2 expression. Significance was calculated by log-rank test. „Patients at risk“ indicates the number of patients who are alive in each group (TGM2 expression weak, moderate or strong) at the corresponding time point (starting point, 2, 4, 6, 8, 10 and 12 years of follow up).

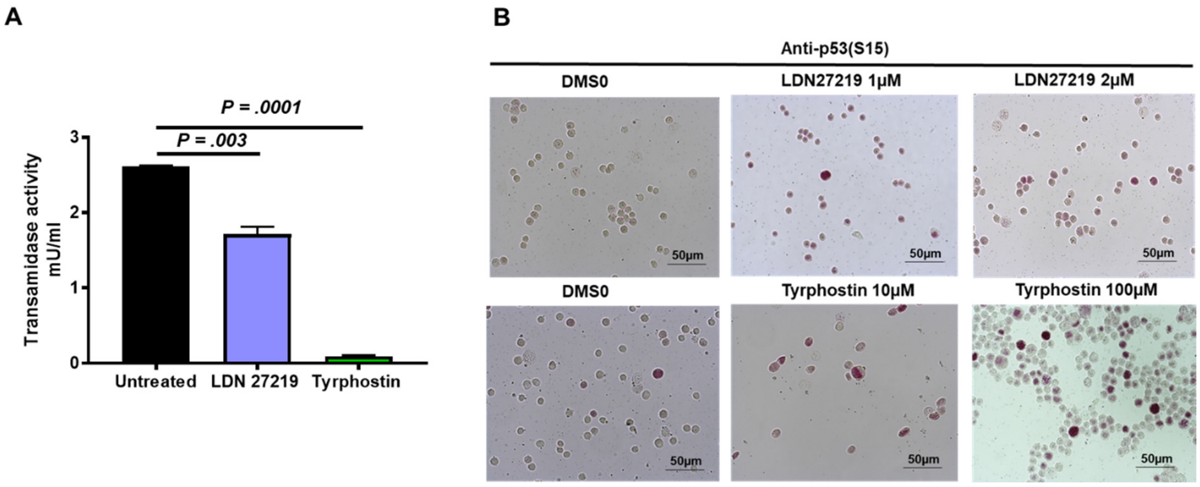

Supplementary Figure 7. TGM2 inhibition results in a significant decrease of transamidase activity and an increase in phosphorylation of p53. (A) TGM2 transamidation activity in SW480 cells after treatment with LDN27219 or Tyrphostin 47. Results are presented as mean ± SD. Significance determined using Multiple t-test or Mann-Whitney-U test. (B) Representative microphotographs of IHC stainings of p53(S15) (red) of SW480 cell treated with LDN27219 (1µM or 2µM), Tyrphostin (10µM or 100µM) or DMSO for 72 hours. Scale bar, 50 µm.

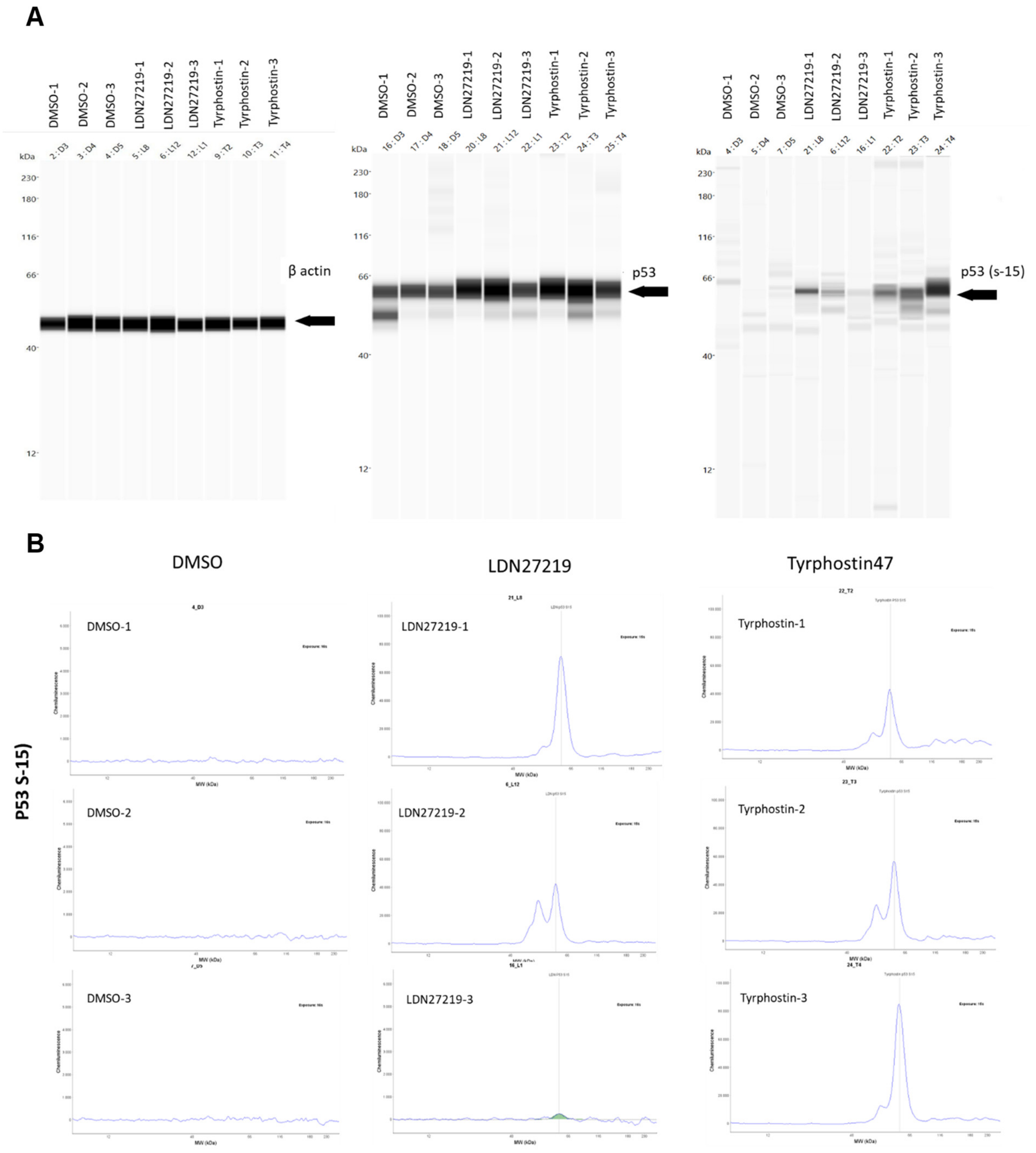

Supplementary Figure 8. Detection of β-Actin, p53 and p53(S15) in explanted tumor xenografts after treatment with DMSO, LDN27219 or Tyrphostin 47 using capillary Simple Western (WES) assay. (A) Results were shown as software-generated lane view images for β-Actin, p53 and p53(S15). All samples were simultaneously analyzed in one run and exposure time was 15 to 16 seconds. (B) Corresponding electropherograms for p53(S15) showing signal intensity and area of the chemiluminescent signal in all 9 samples.

Supplementary Materials and Methods

Clinical tissue samples

A total of 279 patients with CRC were enrolled and underwent surgery at the University Hospital Frankfurt between January 2008 and July 2018. Tumor tissue samples of all patients were obtained from the biobank of the Goethe University Frankfurt Cancer Center's Tissue Procurement Facility. The use of the samples for research purposes was approved by the institutional ethics review board and all patients gave written consent before operation. Tumor samples were dissected and identified by two pathologists to confirm the pathologic nature and histologic grading of the tumor. The surgical specimens were fixed in formalin. Most patients were followed-up every 3-6 months after surgery, including tumor marker testing (serum carcinoembryonic antigen, CEA) and imaging (abdominal ultrasonography, chest radiography or computed tomography). Postoperative adjuvant chemotherapy was recommended in patients with stage III and in stage II tumors with risk factors, according to the German Colorectal Cancer Society Guidelines. Clinicopathological features were evaluated according to the International Union for Cancer Control Tumor Nodular Metastasis Classification (TNM Classification of Malignant Tumors, 7th Edition). The study was approved by the institutional ethics review board (Number: SGI-04-2014). Written informed consent was obtained from all participants prior to inclusion in the study in accordance with the Declaration of Helsinki and local laws and regulations.

Slide digitalization and production of a human tissue micro array (TMA)

Hematoxylin and eosin stains were digitalized using a 20x brightfield slide scanner (Pannoramic Scan II, 3D Histech, Budapest, Hungary). Annotations of defined regions of interest were set to a core diameter of 1 mm. The TMA was produced using the TMA Grand Master (3DHistech, Budapest, Hungary) and matching settings of 1 mm core diameter. Via

slide overlay function, digital annotations were matched to the donor tissue and transferred to an empty recipient paraffin matrix. In summa, three TMAs were produced, each containing well characterized areas of the tumor and tumor adjacent physiological tissue.

Immunohistochemistry (IHC)

To assess TGM2 expression in tumor and adjacent normal colon mucosa, immunohistochemical stainings on the constructed tissue microarrays were performed. After deparaffinization, antigen retrieval was performed by Aptum 2100-Retriever using One R-Universal buffer (Aptum Biologics Ltd., Southampton, UK) for 30 minutes. Subsequently sections were blocked and incubated with a monoclonal mouse anti-human TGM2 antibody (1:100, clone CUB7402, Abcam, Cambridge, UK) and polyclonal anti-p53S15 antibody (1:100, #AF1043, R&D Systems, Minneapolis, USA) for 1 hour. The signal was detected using Multiview (mouse-HRP/rabbit-AP) IHC kit (Enzo Life Sciences, Lörrach, Germany) according to the manufacturer's protocol. TGM2 was visualized using DAB (3,3'-Diaminobenzidine) chromogen (brown) while p53S15 was visualized using AP (Alkaline phosphatase) chromogen (red). The specificity of the monoclonal TGM2 antibody has been confirmed before (15). To confirm the specificity of the antibody against p53(S15) we stained wild type HCT-116 colon cancer cells as well as p53 knockdown HCT-116 cells. There was a specific nuclear p53(S15) staining in wild type cells, while p53 knockout cells showed no staining. (Supplementary Fig. S1). Further, an isotype control was used to exclude unspecific antibody binding. All sections were counterstained with hematoxylin. All slides were reviewed independently by two investigators, blinded to tumor stage and patient outcome. The staining intensity was divided into five groups: no or weak staining (score=1), weak to moderate staining (score=2), moderate staining (score=3), moderate to strong staining (score=4), strong staining (score=5). The proportion of positive cells was grouped into three categories: 0-25% (score=1), 26-75% (score=2) and 76-100% (score=3). The total scores were derived by multiplying the staining

intensity and proportion of positive cell scores. For correlation analysis, staining scores were summarized as weak expression (total score 0-6), moderate expression (total score 7-11) and strong expression (total score 12-15).

Isolation of primary cells from patient specimens

Fresh human colon cancer and adjacent normal mucosa tissue were obtained from patients undergoing surgical resection at Goethe University Hospital Frankfurt or at Bethanien-Hospital (Frankfurt, Germany), who had given informed consent. All tissues were collected under protocols approved by the ethics committee of the University Hospital Frankfurt. All samples were characterized by a pathologist. Briefly, solid tissues were minced into small fragments, washed with PBS containing penicillin/streptomycin followed by enzymatic dissociation with 200 U/ml Collagenase type III, 100 U/ml Dispase and 100 U/ml DNase I (all Worthington Biochemical Corp., Lakewood, USA). During enzymatic incubation, cell suspension was subjected to MACS tissue-dissociator (Miltenyi Biotec, Bergisch-Gladbach, Germany) under a defined program every 15 minutes. The digested material was filtered and contaminating red blood cells were removed by osmotic lysis using 0.83% ammonium chloride solution with 0.1mM EDTA for 10 minutes at 37°C. Magnetic cell separation was performed to obtain an epithelial cell-enriched suspension using the human Tumor Cell Isolation Kit from Miltenyi Biotec (Germany) according to the manufacturer‘s instructions. After magnetic sorting, the viability was assessed using trypan blue exclusion and purified cells were resuspended in serum-free DMEM/F12 (Gibco Fisher Scientific, Schwerte, Germany) supplemented with 20 ng/ml epidermal growth factor (order code # E9644-.2MG) and fibroblast growth factor (order code #F0291-25UG, both purchased from Sigma Aldrich, Taufkirchen, Germany), 2 % N2 supplement (Thermo Fisher Scientific Inc, Waltham, MA, USA), 20 mM HEPES, and 50 U/ml penicillin/streptomycin. The purity of the isolated cells was verified by flow cytometry.

Cell lines and cell culture

The human colorectal cancer cell lines SW480, CaCo2 and HCT-116 were obtained from CLS Cell Lines Service GmbH (Eppelheim, Germany). The p53 knockout colon cancer cell line HCT-116 ($p53^{-/-}$) was obtained from Accegen Biotechnology (Köln, Germany). Cells were cultured in McCoys medium (Gibco Fisher Scientific) containing 10% fetal bovine serum (Gibco Fisher Scientific), 200 mM Hepes, 2 mmol L-glutamine (both Sigma Aldrich), and 100 µg/ml Gentamycin sulfate (Biozym Scientific, Hessisch Oldendorf, Germany). The cells were cultured at 37°C in a humidified atmosphere with 5% CO_2. Authentication of SW480, CaCo2 and HCT-116 cell lines was performed by short tandem repeat (STR) genotyping (CLS Cell Lines Service). All cell lines were mycoplasma free during the course of the experiment. All experiments were performed using cell lines which had been passaged <25 times.

Sphere formation assay

Cancer cell line cells or freshly isolated CRC cells were cultured in serum-free medium described above at a density of 5 000 cells per well in ultra-low-attachment 24-well plates (Corning Inc., Corning, NY, USA). Plates were scored microscopically after 7 and 14 days using Axio Observer Z-1 microscope (Carl Zeiss, Jena, Germany).

In vitro drug treatment

LDN27219 and Tyrphostin 47 (both Sigma Aldrich, St. Louis, USA) were dissolved in dimethyl sulfoxide (DMSO) and stored at -20°C. 3 000 SW480 and CaCo2 cells per well in 96-well plates were cultured with or without LDN27219 (1 or 10µM) or Tyrphostin 47 (10 or 100µM) for 24-72 hours. Proliferation was assessed using 3-(4,5-dimethylthiazol-2yl)-2,5-diphenyltetrazoliumbromide (MTT) assay. Further, the effect of TGM2 inhibition on tumorsphere formation was investigated in SW480, CaCo2 and primary CRC cells using sphere formation assay. Apoptosis was determined using AnnexinV/7AAD staining (BD Becton

Dickinson, Heidelberg, Germany), according to the manufacturer's instructions in SW480 and CaCo2 cells after treatment with LDN27219 and Tyrphostin for 72 hours. In order to determine p53 activity under TGM2 inhibition, phosphorylated p53(S15) was detected by IHC and flow cytometry. For IHC, SW480 cells were cultured in 8-well chamber slide (Corning) and treated with LDN27219 or Tyrphostin for 24 to 72 hours. Subsequently, cells were washed and fixed with 10% formalin solution (Sigma-Aldrich). IHC staining with phosphorylated p53(S15) antibody was performed as described previously in the section Immunohistochemistry. Finally, flow-cytometric assessment of phosphorylate p53(S15) was performed. SW480 cells treated with LDN27219 or Tyrphostin for 24 to 72 hours, were harvested and fixed with 4% formaldehyde, followed by permeabiliziation with ice-cold methanol for 1 hour. Subsequently, cells were washed and stained with FITC-conjugated anti-p53S15 antibody for 1 hour at room temperature. The cells were analyzed on a FACSCanto II (Becton Dickinson, Heidelberg, Germany). DMSO treated cells served as control.

In vivo xenograft experiments

All animal experiments were performed according to protocols approved by the state government. NOD.CB17-*Prkdc*^{scid}/J (NOD-SCID) mice (Jackson Laboratory, Maine, USA) were used for experiments at 6-8 weeks of age. LDN27219 and Tyrphostin 47 (both Sigma Aldrich, St. Louis, USA) were dissolved in dimethyl sulfoxide (DMSO).

5x10^4 living SW480 cells were subcutaneously injected into the flank of NOD-SCID mice. When tumor size reached 0.2-0.3 cm in diameter, LDN27219 (25 mg/kg) was administered orally and Tyrphostin 47 (2,2mg/kg) intraperitoneally three times a week and compared with a DMSO control. Tumor growth was measured twice weekly using a caliper, and mice were sacrificed when tumor size reached a diameter of 1.0 cm. Tumors were harvested and lysates were prepared for transglutaminase activity assay and protein expression analysis by Simple Western technology.

Transglutaminase activity assay

Transamidase activity of TGM2 was assessed in SW480 cells treated with LDN27219, Tyrphostin 47 or DMSO as well as in corresponding xenograft tumors using Tissue Transglutaminase Microassay kit (order code # T055, Zedira, Darmstadt, Germany) following the manufacturer's instructions. Briefly, cells or minced tumor tissue were lysed on ice in M-PER Mammalian extraction Reagent (Thermo Fisher Scientific) and protein concentration was determined subsequently. Protein samples were incubated with reaction buffer containing calcium, DTT and biotin-pepT26 in the wells of the microtiter plate. For negative control, EDTA was added. The assay uses biotin-pep26 as the first substrate and an amine donor/acyl-acceptor as a second substrate. In the presence of active TGM2, the γ-carboxamide of the glutaminyl residue of biotin-pepT26 is incorporated into the amine substrate to form biotinylated isopetide bound. Enzymatic reaction is determined by its interaction with streptavidin labelled peroxidase. A substrate solution for peroxidase was added for color development. The color intensity was measured on the Infinite 200 microplate reader (Tecan, Germany).

Protein expression analysis by Simple Western technology

Protein expression was detected by Simple Western™ assays using the Wes™ System following the manufacturer's protocol (Bio-Techne, Wiesbaden, Germany). The automated capillary western blot assay incorporates protein separation based on size performed in glass capillaries and immobilization of the proteins directly onto the capillary walls, followed by immuno-probing and chemiluminescent detection. This method was used to analyze protein expression in tumor lysates. Briefly, tumor tissues were lysed by m-PER mammalian extraction buffer containing 1x Halt protease inhibitor (Thermo Fisher Scientific) and homogenized by Precellys homogenizer (Peqlab Biotechnologie, Erlangen, Germany). Protein concentration was determined using Coomassie plus (Bradford) assay (Thermo Fisher Scientific). 0.05 µg

tumor lysates were diluted with 0.1 × Sample Buffer, combined with Fluorescent Master Mix (containing sample buffer, fluorescent standard, and DTT) and denaturated at 95 °C for 5 minutes. The denatured samples, blocking reagent, primary antibodies, HRP-conjugated secondary antibodies and chemiluminescent substrate were then applied on a Protein Simple 12-230 kDa capillary cartridge separation module (Bio-Techne). The primary antibody dilution was 1:100 to 1:50. The following primary antibodies were used: anti-p53 (clone 1C12, #2524) and anti-phospho-p53(S15) (clone 16G8, #9286, both Cell Signaling Technology, Danvers, Massachusetts, USA). Beta Actin (#MAB8929, Cell Signaling Technology) served as loading control. A biotinylated ladder provided molecular weight standards for each assay. After plate loading, the separation electrophoresis and immunodetection steps take place in the fully automated capillary system. An optimized defined run setting was used. Protein expression was measured at exposure times between 15 and 16 seconds. Quantification of chemiluminescence was based on peak area after correction for a baseline signal. Molecular weight, the area under the peak, signal-to-noise ratio, peak height, and peak width were reported for each named peak. The area under the peak represents the signal intensity of the immuno-detected protein. Data were generated by the Compass software for Simple Western instruments.

Statistical analysis

The level of TGM2 expression in CRC and normal colorectal mucosa, as well as the relationship between TGM2 expression level and clinicopathological factors were analyzed using Wilcoson's matched paired and Chi-squared (χ^2)-test.

For baseline characteristics the follow-up started with the date of first diagnosis. End of follow-up was the date of death or the last patient visit. Categorical variables were described in frequencies and percentages. Continuous variables were represented as a mean and its standard deviation (SD). Categorical variables were compared by the Chi-squared (χ^2)-test or Fisher's exact test, as needed. χ^2 – Test was performed over three groups of TGM2 expression.

Continuous variables were compared using One-way analysis of variance (ANOVA). For measurement of rank correlation between UICC stage and TGM2 expression Spearman's rank correlation analysis was used. Spearman's rank correlation coefficient as a measurement of the effect size was interpreted according to Cohen. Overall Survival, Disease-free Survival and Progression-free Survival were estimated using a Kaplan-Meier survival analysis considering the time to death or disease relapse/progression in months. Survival curves were generated using the Kaplan-Meier method and differences in survival were tested with the log-rank test. Factors potentially associated with overall mortality and risk of progression were analyzed using the Cox Proportional Hazards model with stepwise selection. A univariate Cox Proportional Hazards model analysis was performed with all variables, and those with a P-value <0.05 were used for multivariate analysis. UICC status was excluded in multivariate analyses to avoid multicollinearity. Results were expressed as a hazard ratio (HR) with a 95% confidence interval (CI).

We analyzed in vitro and in vivo assay results using multiple t-test or Mann-Whitney-U test. All statistical analyses were performed in GraphPad Prism 6 or International Business Machines Corporation (IBM) Statistical Package for the Social Sciences (SPSS) for Windows (version 22.0; IBM, Chicago, IL, USA). A P-value <0.05 was considered statistically significant in all tests.
